# LncRNA NEAT1/miR-129/Bcl-2 signaling axis contributes to HDAC inhibitor tolerance in nasopharyngeal cancer

**DOI:** 10.18632/aging.103427

**Published:** 2020-07-21

**Authors:** Fei Xue, You Cheng, Li Xu, Chuan Tian, Hongye Jiao, Rui Wang, Xia Gao

**Affiliations:** 1 Department of Otorhinolaryngology-Head and Neck Surgery, Affiliated Jinling Hospital, Medical School of Nanjing University, Nanjing 210002, Jiangsu, China; 2 Department of Medical Oncology, Affiliated Jinling Hospital, Medical School of Nanjing University, Nanjing 210002, Jiangsu, China; 3 Department of Otorhinolaryngology-Head and Neck Surgery, Affiliated Drum Tower Hospital, Medical School of Nanjing University, Nanjing 210008, Jiangsu, China

**Keywords:** lncRNA NEAT1, Bcl-2, HDAC inhibitor, NPC

## Abstract

Histone deacetylase inhibitors (HDACis) - based therapeutic drug tolerance is one of the principal factors of poor prognosis of patients with nasopharyngeal cancer (NPC). Mechanisms of tolerance to HDACis are not well understood. Nowadays, dysregulation of long non-coding RNAs (LncRNAs) and microRNAs (miRNAs) has been reported to provide beneficial or inhibitory effects in drug-tolerance in various cancers. Herein, we established the HDAC inhibitor (SAHA)-tolerant NPC cell sublines, which had decreased apoptosis in response to SAHA treatment. We observed that the expression of miR-129 was significantly reduced in SAHA-tolerant NPC cells. Manipulating the expression of miR-129 overcame SAHA tolerance, and enhanced the SAHA-induced apoptosis. In terms of miR-129 downregulation, we identified that NEAT1 suppresses miR-129 expression. NEAT1 was found to be upregulated in SAHA tolerance cells. The depletion of NEAT1 phenocopied the effect of miR-129 overexpression, which also enhanced SAHA-induced apoptosis. Bcl-2 was the downstream target of miR-129 and contributed to SAHA tolerance in NPC. Our in vivo xenograft experiment confirmed that the administration of miR-129 or inhibition of Bcl-2 overcame the SAHA tolerance in NPC. In conclusion, NEAT1 increases in NPC tissues and manages to facilitate SAHA tolerance by modulating the miR-129/Bcl-2 axis, providing novel therapeutic targets for NPC treatment.

## INTRODUCTION

Nasopharyngeal cancer (NPC) is a disease where malignant tumor locates at the nasopharynx mucosa epithelium. The nonkeratinizing undifferentiated NPC is a particularly prevalent type of carcinomas in the south of China, which is closely related to the Epstein–Barr virus (EBV) infections [1]. Due to the obscure symptoms and aggressiveness, over 70% of NPC patients are likely to be diagnosed in a very late stage, which results in unfavorable prognosis [2, 3]. Although chemotherapy and radiotherapy are two major treatment options, neoadjuvant therapy is currently applied more frequently [2]. Epigenetic changes have been confirmed to play an essential role in the maintenance and evolution of cancer. Modulation of the acetylation status of histones is an important mechanism for regulation of genes’ expression and is directly associated with the activity of histone acetyltransferases and histone deacetylases (HDACs) [4]. Therefore, targeting HDACs through histone modification can be a potent and efficient adjuvant treatment for NPC.

HDACs are epigenetic enzymes that can modulate gene expression by eliminating the acetyl group from histones, which make them as well-established targets in anticancer therapy [5]. More and more HDAC inhibitors (HDACis) are being designed and tested in Phase I, II, and III clinical trials [6]. Several HDACis, such as suberoylanilide hydroxamic acid (SAHA; Vorinostat), have been approved for treating hematopoietic malignancies [5, 7]. However, HDACi as single agents are generally ineffective for solid tumors, and tolerance to HDACi represents a major obstacle in their clinical applications [7, 8]. The precise mechanism underlying HDACis treatment failure remains unclear and needs further investigation.

Certain studies have indicated that microRNA (miRNA) dysregulation can facilitate HDACi tolerance in multiple cancers [9, 10]. MiRNAs inhibit gene expression by connecting with complementary sequences in the 3' region of mRNAs, and subsequently degrade mRNA or hinder translation [11]. The miRNA hybridization with mRNAs degrades mRNA, suppresses translation, and consequently modulates various cellular processes [12]. HDACi can quickly change miRNA levels and trigger cell death [13, 14]. We speculated that defects in apoptotic pathways mediated by miRNA could result in HDACi tolerance in NPC.

Recently, miR-129, an anti-tumor miRNA, was reported to be necessary for HDACi-induced cell death [14, 15]. However, the precise effect and function of miR-129 on HDACi tolerance of NPC needs further investigation. In this study, we studied the mechanism of HDACi resistance using suberoylanilide hydroxamic acid (SAHA), a pan HDACs inhibitor. We found that miR-129 expression was downregulated in SAHA-tolerant NPC cells. The long non-coding RNA (LncRNA), NEAT1, was found to sponge miR-129 and suppressed its mRNA level in resistant NPC cells. Moreover, we identified that Bcl-2 was the target of miR-129 that mediates SAHA tolerance in NPC cells. Furthermore, we confirmed that rescuing the miR-129 level or inhibition of Bcl-2 re-sensitized NPC-tolerant cells to HDACi. Hence, targeting molecules specific to NEAT1/miR-129/Bcl-2 inside tumor cells can be a novel potential cancer treatment compared to conventional chemotherapy.

## RESULTS

### SAHA-tolerant NPC cells exhibit less apoptosis

**Figure 1:**
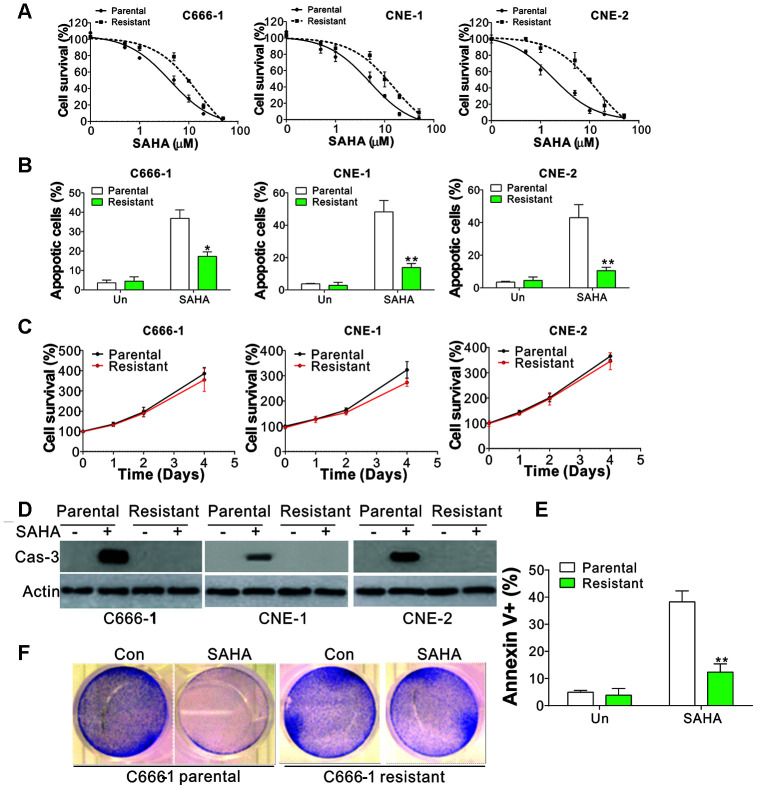
**SAHA-tolerant NPC cell exhibits apoptosis defects.** (**A**) The cell viability in parental and SAHA-tolerant phenotype upon different concentrations of SAHA treatment for 24 h was analyzed by MTS assay. (**B**) The apoptosis of parental and SAHA-tolerant phenotype subjected to 4 μmol/L of SAHA for 24 h. (**C**) The proliferation of parental and SAHA-tolerant phenotype was analyzed by MTS assay upon different time points. (**D**) The cleaved Cas-3 expression in parental and SAHA-tolerant phenotype subjected to 4 μmol/L of SAHA for 24 h. (**E**) The parental and SAHA-tolerant C666-1 cells were subjected to 4 μmol/L of SAHA for 24 h, and stained with Annexin V and PI, followed by flow cytometry analysis. (**F**) The crystal violet staining of parental and SAHA-tolerant C666-1 cells was subjected to 4 μmol/L of SAHA for 24 h. Each experiment was performed for 3 times. *, p<0.05; **, p<0.01.

To investigate the mechanism underlying drug tolerance in NPC, we generated three SAHA-tolerant NPC cell lines, including C666-1, CNE-1, and CNE-2. The tolerant cells (-R cells) showed higher IC50 values compared to the parental cells (-P cells) (Figure 1A). As SAHA suppressed NPC growth by inducing apoptosis [16], we studied the apoptosis induced by SAHA in NPC parental and tolerant cells through nuclear Hoechst 33258 staining. We then observed that the SAHA-tolerant cell lines exhibited lower levels of apoptosis when compared with the parental lines (Figure 1B). In contrast, there was no significant difference in cell proliferation between NPC parental and tolerant cells (Figure 1C). Furthermore, the expression of cleaved caspase-3 (Cas-3) and the caspases 3/7 activity was investigated to study the apoptosis induced by SAHA in parental and resistant NPC cells. SAHA treatment led to the caspase-3 cleavage and activation of caspases 3/7 in NPC parental cells, which did not occur in NPC-tolerant cells (Figure 1D, Supplementary Figure 1A). Moreover, flow cytometry and crystal violet staining further supported that SAHA induced lower levels of apoptosis in NPC-tolerant cells (Figure 1E, 1F). Since SAHA is a pan HDAC inhibitor, we further tested whether SAHA resistant cells also take effects on other specific HDAC inhibitors. We found that C666-1R cells were substantially resistant to HDAC1-3 inhibitor, MS-275, and HDAC8 inhibitor, PCI34051, but remained sensitivity to HDAC6 inhibitor, Cay10603 (Supplementary Figure 1B). These results suggested that the effect of SAHA in NPC should be correlated to HDAC1-3, and 8, which is consistent with a previous study showing that HDAC6 is not necessary for HDACi induces apoptosis of NPC cells [17]. Collectively, our data suggest that abnormal apoptosis is the major mechanism for HDAC inhibitor tolerance in NPC cells.

### Suppression of miR-129 mediated the SAHA tolerance in NPC cells

**Figure 2:**
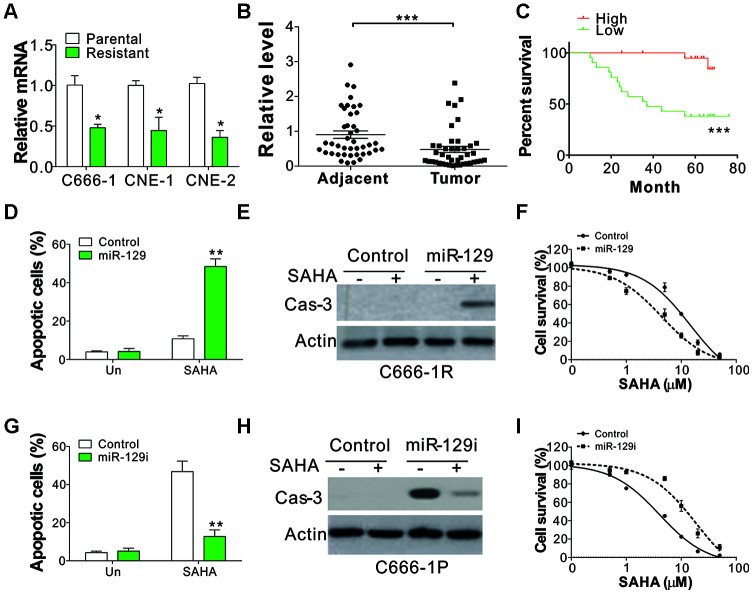
**miR-129 downregulation contributes to SAHA tolerance in NPC.** (**A**) The mRNA level of miR-129 in parental and SAHA-tolerant phenotype. (**B**) The relative level of miR-129 in 42 pair of adjacent tissue and primary NPC tumors. The Y-axis is on a linear scale. (**C**) The survival of NPC patients with different levels of miR-129. If the expression level was higher than the average value of miR-129 expression in primary tumors, the patients were grouped into high expression group (n=15). The left patients were grouped as miR-129 low expression patients (n=27). (**D**) C666-1 cells were subjected with control or miR-129 mimic, followed by 4 μmol/L of SAHA treatment for 1 d. The apoptosis was investigated through HOECHST 33258 staining. (**E**) The cleaved caspase-3 in C666-1 cells treated in (**D**). (**F**) The survival of C666-1 cells transfected with control or miR-129 mimic, followed by different concentrations of SAHA treatment. (**G**) C666-1 cells were transfected with control or miR-129 antagomir, followed by 4 μM SAHA treatment for 24 h. The apoptosis was analyzed through HOCHST 33258 staining. (**H**) The cleaved caspase-3 in C666-1 cells treated in (**G**). (**I**) The survival of C666-1 cells transfected with control or miR-129 antagomir, followed by different concentrations of SAHA treatment. Each experiment was performed for 3 times. *, p<0.05; **, p<0.01; p<0.001.

MicroRNA was assumed to be a crucial component in mediating drug tolerance in multiple cancers, and miR-129 was found to be a prerequisite for HDACi-induced cell death [14, 15]. Therefore, we studied the regulation of miR-129 in NPC parental and tolerant cells. As predicted, miR-129 expression level decreased in NPC-tolerant cells (Figure 2A). Subsequently, we studied miR-129 expression level in 42 pairs of adjacent tissues and primary tumors from NPC patients, and found that miR-129 expression level was lower in the NPC tumor samples (Figure 2B). The low expression of miR-129 was also correlated to poor survival rates of NPC patients (Figure 2C), suggesting the tumor-suppressive role of miR-129. To thoroughly explore the role of miR-129 in HDAC tolerance, we transfected the SAHA-tolerant C666-1 cells (C666-1R) with miR-129 mimic. We found that recovering the expression of miR-129 in C666-1R cells re-sensitized the cell to SAHA induced apoptosis (Figure 2D, 2E). Accordingly, IC50 levels decreased following miR-129 mimic transfection in C666-1R cells (Figure 2F). Reversely, transfection of miR-129 antagomir in C666-1 parental cells (C666-1P) suppressed SAHA-induced apoptosis (Figure 2G, 2H), and hence increased the IC50 value of SAHA (Figure 2I). These findings displayed that NPC cells acquired tolerance to SAHA via miR-129 downregulation.

### NEAT1 sponged miR-129 in SAHA-tolerant NPC cells

**Figure 3:**
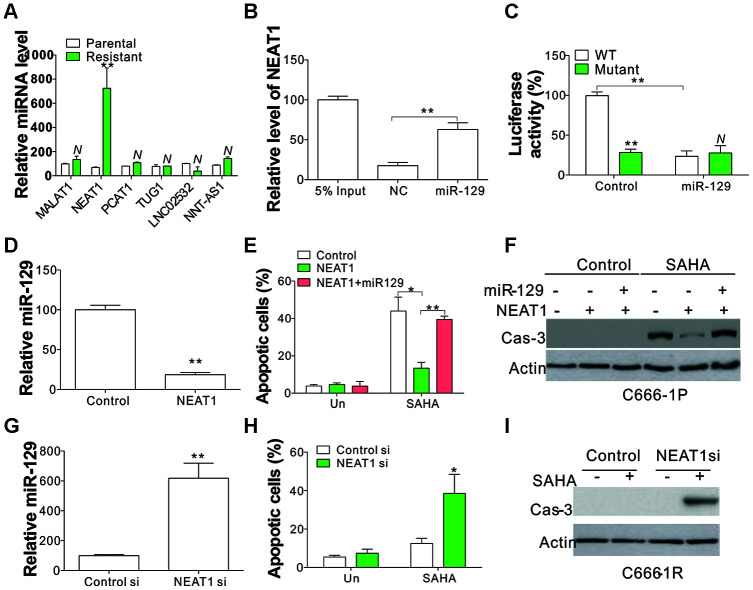
**Mir-129 is the sponging target of NEAT1.** (**A**) The expression of indicated LncRNA in parental and SAHA-tolerant C666-1 cells. (**B**) A specific biotin-marked miR-129 probe to successfully arrest NEAT1 in relation to NC group. NC was referred to the non-specific biotin-labeled probe. (**C**) DLR assay was conducted to identify the direct link of NEAT1 and miR-129 on the basis of their complementary sequences. The pMIR-REPORT constructs with WT and mutant NEAT1 fragment insert were used. (**D**) The miR-129 expression in C666-1 cells with lenti-NEAT1. (**E**) C666-1 cells were treated with control or NEAT1 plasmid with or without co-transfection of miR-129 mimic, followed by 4 μmol/L of SAHA treatment for 1 d. The apoptosis was investigated through HOECHST 33258 staining. (**F**) The cleaved Cas-3 in C666-1 cells treated in (**E**). (**G**) The miR-129 expression in C666-1 cells transfected with NEAT1 siRNA. (**H**) C666-1 cells were treated with control or NEAT1 siRNA, followed by 4 μmol/L of SAHA treatment for 1 d. Apoptosis was investigated through HOECHST 33258 staining. (**I**) The cleaved Cas-3 in C666-1 cells treated in (H). Each experiment was performed for 3 times. N, p>0.05; *, p<0.05; **, p<0.01.

LncRNAs may serve as molecular sponges because they harbor binding sites for miRNAs and sequester them away from their mRNA targets, which might contribute to drug tolerance in different cancer types [18]. We noticed that miR-129 was reported to be modulated by several LncRNAs, including MALAT1, NEAT1, PCAT1, TUG1, LNC02532, and NNT-AS1. Hence, we studied the regulation of these LncRNAs in three pairs of NPC parental and resistant cells and found that only NEAT1 was consistently upregulated in resistant cells (Figure 3A, Supplementary Figure 2), indicating that NEAT1 might suppress the miR-129 expression in SAHA-tolerant NPC cells. To further verify the interaction of miR-129 and NEAT1, a specific biotin-labelled miR-129 probe successfully captured more NEAT1 than the non-specific biotin-labeled oligo probe (NC group) (Figure 3B). We also performed a dual-luciferase reporter assay to determine the direct binding between NEAT1 and miR-129 based on their complementary sequences. A NEAT1 wild type (WT) or mutant fragment was inserted into luciferase reporter. Then, we co-transfected a miR-129 mimic with the reporter gene into C666-1 cells. A significant reduction in WT luciferase reporter activity was observed relative to co-transfection with miR-129 mimic (Figure 3C), which was absent in mutant luciferase reporter transfection (Figure 3C), thus confirming the direct interaction of NEAT1 and miR-129. Consequently, we investigated the expression level of miR-129 in NEAT1 overexpressing C666-1P cells, and found that NEAT1 expression suppressed the miR-129 level (Figure 3D). Consistently, NEAT1 overexpression in C666-1P cells suppressed SAHA-induced apoptosis (Figure 3E, 3F). The Co-transfection of miR-129 mimics compromised the inhibitory effects of NEAT1 overexpression on SAHA-induced C666-1P apoptosis (Figure 3E, 3F). On the other hand, depletion of NEAT1 by its siRNA increased the expression of miR-129 in C666-1R cells (Figure 3G), and re-sensitized the tolerant cells to SAHA-induced apoptosis (Figure 3H, 3I). Collectively, our results indicate that induction of NEAT1 in SAHA-tolerant NPC cells suppressed the miR-129 expression level, and contributed to SAHA tolerance in NPC cells.

### BCL2 is a direct target of miR-129 in NPC cells

**Figure 4:**
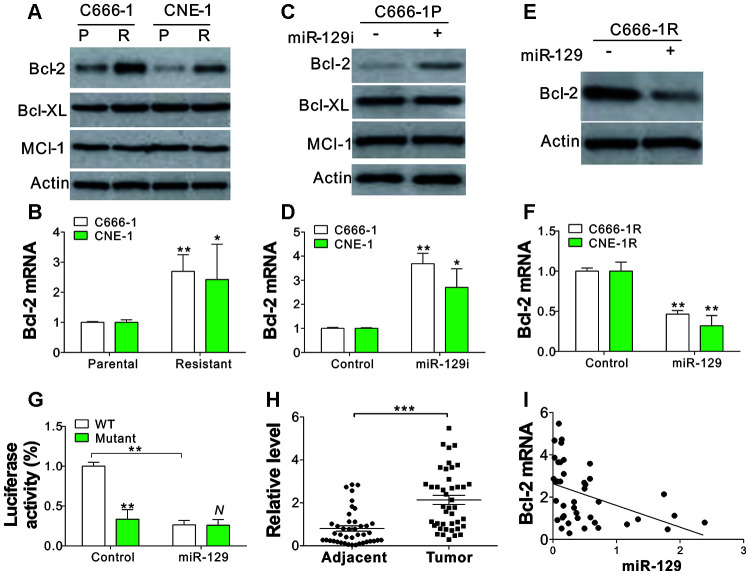
**Bcl-2 is the target of miR-129 in SAHA-tolerant NPC cells.** (**A**) The WB of indicated proteins in SAHA-tolerant C666-1 and CNE-1 cells. (**B**) The mRNA level of Bcl-2 in SAHA-tolerant C666-1 and CNE-1 cells. (**C**) The expression of indicated proteins in C666-1 cells treated with miR-129 antagomir. (**D**) The Bcl-2 mRNA level in C666-1 and CNE-1 cells transfected with miR-129 antagomir. (**E**) The Bcl-2 expression in SAHA-tolerant C666-1 cells treated with miR-129 mimic. (**F**) The Bcl-2 mRNA level in SAHA-tolerant C666-1 and CNE-1 cells treated with miR-129 mimic. (**G**) The Bcl-2 reporter luciferase activity in C666-1 cells treated with miR-129 mimic. (**H**) The Bcl-2 mRNA level in 42 pair of adjacent tissues and primary NPC tumors. The Y-axis is on a linear scale. (**I**) The correlation of Bcl-2 and miR-129 expression in 42 NPC patient tumors. R^2^=0.1816. Each experiment was performed for 3 times. N, p>0.05; *, p<0.05; **, p<0.01; p<0.001.

BCL2 family proteins are essential molecules for apoptosis regulation that have been reported to be the target of miR-129 [19]. Therefore, we investigated the regulation of several anti-apoptotic Bcl-2 family proteins in NPC parental and tolerant cells. We observed that Bcl-2 in C666-1 and CNE-1 tolerant cells was upregulated (Figure 4A). However, Bcl-XL and Mcl-1 expression did not have any changes in C666-1 and CNE-1 parental and tolerant cells (Figure 4A). Bcl-2 upregulation in NPC-tolerant cells also occurred in their mRNA level (Figure 4B), indicating the transcriptional level changes of Bcl-2. TargetScan and RNAhybrid algorithms have been used to identify a predictive miR-129-binding site at 1525–1531 positions in BCL2 mRNA 3′-UTR [19]. To confirm that miR-129 regulated bcl2 expression, we transfected C666-1P cells with miR-129 antagomir and tested the expression of Bcl-2 family proteins on protein and mRNA level. We observed that miR-129 inhibition increased Bcl-2 expression in protein and mRNA level (Figure 4C, 4D), but did not alter the expression of Bcl-XL and Mcl-1 (Figure 4C). On the other hand, miR-129 mimic transfection in C666-1R cells suppressed the expression of Bcl-2 on protein and mRNA level (Figure 4E, 4F). Furthermore, we co-transfected the WT Bcl-2 luciferase reporter vector with miRNA or miR-129 into C666-1 cells and found that miR-129 significantly suppressed luciferase activity (Figure 4G). However, miR-129 failed to suppress the luciferase activity of the reporter vector with Bcl-2 3’-UTR with three points mutation in miR-129-binding site (Figure 4G). The mRNA level of Bcl-2 was also greater that in the primary NPC tumors when compared with the adjacent tissue (Figure 4H), and was negatively correlative to the expression level of miR-129 (Figure 4I). These findings indicate that miR-129 specifically represses the expression of Bcl-2 protein in NPC cells.

### Suppressing Bcl-2 expression enabled miR-129 to mediate SAHA sensitivity in NPC cells

**Figure 5:**
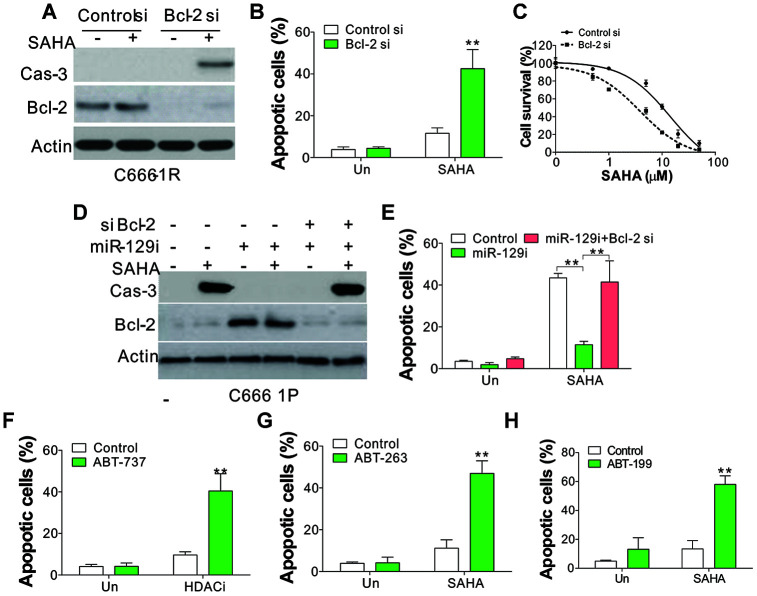
**Bcl-2 mediated the SAHA tolerance in NPC cells.** (**A**) SAHA-tolerant C666-1 cells were transfected with control or Bcl-2 siRNA, followed by 4 μmol/L of SAHA treatment for 1 d. The cleaved Cas-3 expression was investigated via WB. (**B**) The apoptosis of C666-1 cells obtained in line with (**A**). (**C**) The survival of SAHA-tolerant C666-1 cells treated with control or Bcl-2 siRNA, followed by different concentrations of SAHA treatment. (**D**) C666-1 cells were subjected to control or miR-129 antagomir with or without co-transfection of Bcl-2 siRNA, followed by 4 μmol/L of SAHA treatment for 1 d. The cleaved Cas-3 expression was investigated via WB. (**E**) The apoptosis of C666-1 cells obtained in line with (**D**). (**F**) The apoptosis of SAHA-tolerant C666-1 cells treated with 4 μmol/L of SAHA with or without co-treatment of ABT-737 (2 μmol/L). (**G**) The apoptosis of SAHA-tolerant C666-1 cells treated with 4 μmol/L SAHA with or without co-treatment of ABT-263 (2 μmol/L). (**H**) The apoptosis of SAHA-tolerant C666-1 cells treated with 4 μmol/L SAHA with or without co-treatment of ABT-199 (2 μmol/L). Each experiment was performed for 3 times. **, p<0.01.

Subsequently, we examined the function of Bcl-2 in miR-129 mediated SAHA sensitivity in NPC cells. Bcl-2 silence by siRNA in C666-1R cells re-sensitized cells to SAHA-induced apoptosis (Figure 5A–5C), which was similar to the effect of miR-129 mimic transfection. Since miR-129 antagomir was found to suppress the NPC cells sensitivity, we further tested whether Bcl-2 depletion can reverse the effect of miR-129 antagomir or not. We found that miR-129 antagomir suppressed the SAHA induced apoptosis in C666-1P cells, which was reverted by co-transfection of Bcl-2 siRNA (Figure 5D, 5E). Furthermore, Bcl-2 inhibitors, such as ABT-737, ABT-263, and ABT-199, were also found to sensitize C666-1R cells to SAHA-induced apoptosis (Figure 5F–5H). Therefore, our results indicate that Bcl-2 is the target of miR-129 in modulating SAHA sensitivity in NPC cells.

### MiR-129 mediate SAHA sensitivity in NPC *in vivo*

**Figure 6:**
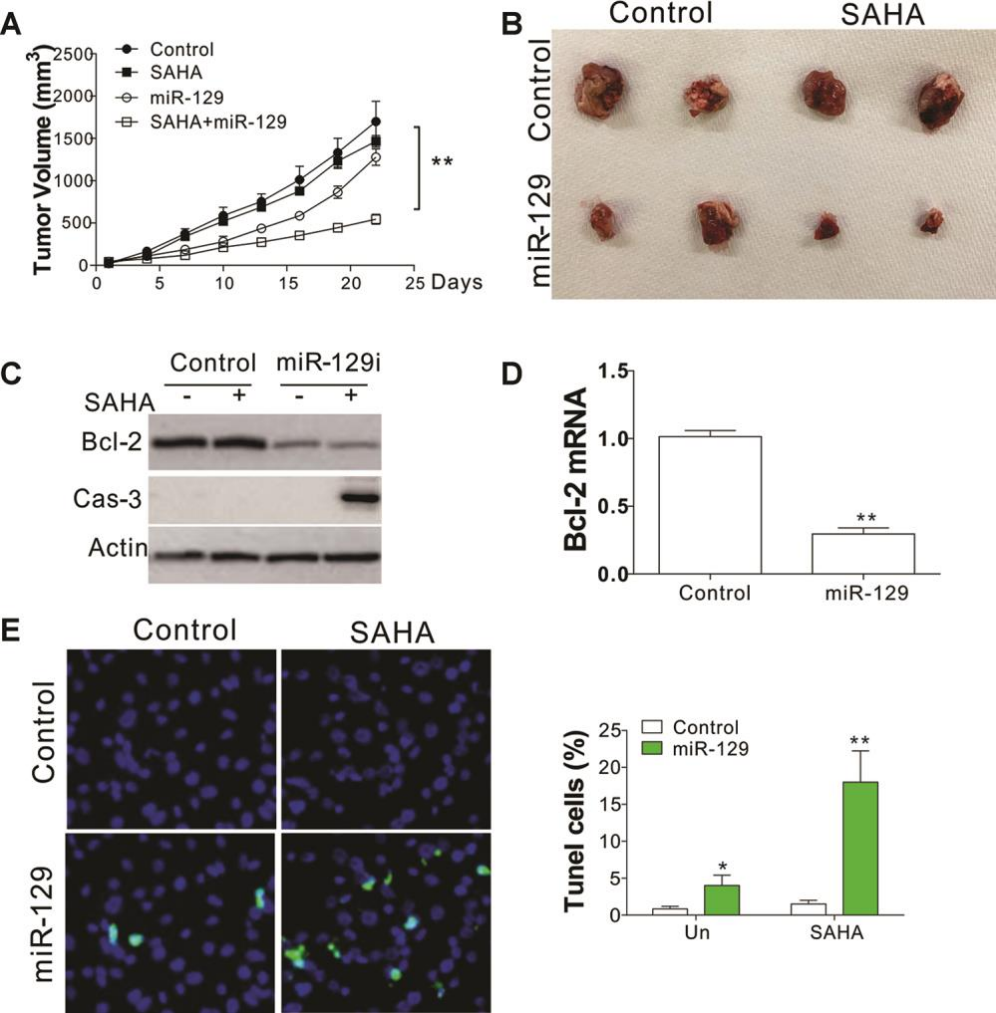
**miR-129 mediated SAHA tolerance *in vivo*.** (**A**) The tumor growth curves of nude mice xenografted with C666-1R cells and treated with SAHA and/or miR-129 mimic. (**B**) The respective tumors. (**C**) The expression of indicated proteins in different groups of tumors. (**D**) The mRNA level of Bcl-2 in tumors. (**E**) The TUNEL staining of tumors in each group. *, p<0.05; **, p<0.01.

To further validate the function of miR-129 in mediating SAHA sensitivity *in vivo*, we implanted SAHA-tolerant C666-1 cells into flanks of BALB/c nude mice. Seven days later, the mice were randomly classified into four groups and were intratumorally injected with miR-129 or control oligo as a negative control (Con), or I.P. injection of SAHA (50 mg/kg/day) or the control vehicle for 14 days. Three weeks after implantation, SAHA treatment had less effect on C666-1R xenografted tumors (Figure 6A, 6B), suggesting that the C666-1R xenografted tumors were tolerant to SAHA *in vivo*. Intratumoral injection of miR-129 in C666-1R xenografted tumor suppressed the tumor growth to a less extent, but significantly enhanced the tumor-suppressive effect of SAHA (Figure 6A, 6B). The western blot and RT-PCR revealed that Bcl-2 expression level was suppressed by miR-129 mimic injection on protein and mRNA level (Figure 6C, 6D). The cleavage of caspase-3 was maximal when treated with miR-129 mimic combined with SAHA treatment (Figure 6C). Furthermore, TUNEL staining displayed that a combined treatment facilitates higher levels of apoptosis in tumors compared to the other three groups (Figure 6E). Similarly, inhibition of Bcl-2 by its specific inhibitor, ABT-199, can also enhance the killing effect of SAHA in C666-1R xenografted tumors as indicated by tumor growth curve (Supplementary Figure 3A) and TUNEL staining (Supplementary Figure 3B). Therefore, the miR-129/Bcl-2 axis mediated the SAHA sensitivity in NPC-tolerant tumor *in vivo*, which might provide a novel therapeutic strategy to overcome HDACi tolerance in NPC cancer.

## DISCUSSION

The tumor-suppressing effects of HDACis are related to the transcriptional change of specific cancer-associated genes, including some critical regulators of the cell cycle, apoptosis, differentiation, angiogenesis, and invasion [20, 21]. As one of the major HDAC inhibitors, SAHA has a broad spectrum of epigenetic processes and facilitates cancer-suppressing activities in numerous solid human tumors [22]. However, tolerance to HDACi commonly occurs in multiple solid cancers, including NPC [23], yet its underlying mechanism remains unclear. miRNAs are a critical type of ncRNA molecules that can regulate post-transcriptional modulation of gene expression in multiple cells, and are involved in multiple drug tolerance during cancer therapy [9]. Herein, we observed that miR-129 was reduced in SAHA-tolerant NPC cells, a fact that directly contributed to drug tolerance. The regulation of miR-129 was controlled by the NEAT1, which was upregulated in SAHA-tolerant NPC cells, and sponged the expression of miR-129. We also identified that Bcl-2, an anti-apoptotic Bcl-2 family protein, was the target of miR-129 that mediates SAHA tolerance in NPC cells. Although the function of NEAT1/miR-129 and miR-129/Bcl-2 axis was studied in multiple cancers [19, 24], it remains unclear their correlation and function in the nasopharyngeal cancer (NPC), especially in term of the HDACi response. Our results collectively suggest that recovery expression of miR-129 can overcome SAHA tolerance in NPC cells, a finding that can provide future therapeutic strategies in NPC treatment.

MiR-129, as a tumor-suppressive miRNA, has been reported to target several oncogenes to prohibit cell proliferation and induce apoptosis in various cancers [25, 26]. However, the exact miR-129 function in NPC progression is yet reported. In our study, we also found that miR-129 acts as a tumor suppressor in NPC, and its dysregulation facilitates HDACi tolerance in NPC. Accumulating evidence has shown that epigenetic aberrations in cancer lead to hypermethylation of multiple genes, including miRNAs, which are crucial in the diagnosis, prognosis, and prediction of the underlying therapeutic efficacy [27, 28]. HDACis are a comparatively new type of anticancer agents that assume essential parts in epigenetic or non-epigenetic modulation, eliciting a series of cancer cells’ physiological processes. HDAC inhibitor treatment can restore the expression of miR-129 in CRC cell lines, which was silenced by DNA methylation [27]. MiR-129 was also reported to be a prerequisite for HDACi-triggered cell death in thyroid cancer cells [14]. Moreover, miR-129 can also enhance the ‘shock and kill’ effect of HDACIs [15]. Our results are also consistent with previous studies in terms of suggesting the miR-129 is essential for the NPC suppression by HDACi, and its downregulation may hinder the apoptosis induced by HDACi, and hence contribute to drug tolerance.

Other than epigenetic regulation, miRNAs were also modified by its interaction with LncRNA [29]. In this study, we analyzed several LncRNA expression profiles and found that NEAT1 acts as the miR-129 sponge that mediates HDACi tolerance in NPC. NEAT1 performs regulatory actions in the majority of cancers by influencing the growth and physiological properties of tumors [24]. Nevertheless, the underlying mechanisms remain thorough investigation. This study reveals that NEAT1 expression was notably augmented in SAHA tolerance NPC cell lines. NEAT1 was found to facilitate hepatocellular carcinoma cell proliferation as a result of its interaction with miR-129 [30]. Consistently, we also found that NEAT1 binds to miR-129, and triggers its downregulation in SAHA-tolerant NPC cells. The decreased NEAT1 exhibits reduced cell viability and increased apoptosis in NPC-tolerant cells upon HDACi treatment. Consequently, we confirmed that high NEAT1 expression suppresses miR-129 expression and contributes to HDACi tolerance in NPC. These results added a novel function of NEAT1 in mediating the HDACi sensitivity in cancer therapy.

HDACi triggers apoptosis of various malignant cells [5]. Downregulation of endogenous Bcl-2 expression is essential for HDACi-induced apoptosis [31]. Consistently, increased Bcl-2 levels have previously been associated with tolerance against chemotherapy of transformed cells and possibly is a critical factor in triggering the tolerance observed with HDACi [32, 33]. However, the underlying mechanism of the effect of Bcl-2 overexpression in HDACi-tolerant tumor still remains unclear. Our study indicates that miR-129 downregulation in HDACi-tolerant NPC cells facilitates Bcl-2 overexpression. Some studies display that the combined use of HDACis and other agents can be particularly advantageous when treating solid tumors [34]. The recent approval of ABT-199 (Venetoclax) makes the suppressing prosurvival Bcl-2 family proteins become a notable treatment approach [35]. In our study, we found that the mechanism of NPC resistant to SAHA is correlated to dysregulation of Bcl-2, an anti-apoptotic protein. Since SAHA was reported to induce apoptosis to kill the cancer cells, the NEAT1/miR-129 mediated abnormal expression of Bcl-2 served as the major cause. Therefore, higher miR-129 can benefit patient survival and tumor suppression, as shown in Figure 2C and Figure 6A. We also found that supplement of miR-129 or inhibition of Bcl-2 by ABT-199 not only lead to cell death in SAHA resistant tumors (Figure 6E, and Supplementary Figure 3B), but also enhanced the killing effect of SAHA. We, therefore, believed that the dysregulation of Bcl-2 by miR-129 suppresses the cell death induced by SAHA contribute to drug resistance. Of course, other factors, such as tumor microenvironment or cell-cell interaction, might also contribute to drug resistance, which needs further investigation.

In summary, epigenetic treatment based on the application of HDACis combined with other targeted therapies may be a new strategy for cancer therapy in the future. As to NPC, the combined use of miR-129 or Bcl-2 inhibitors exhibited great potential in enhancing the killing effect of HDACi in NPC cells. Therefore, restoration of miR-129 levels or targeting NEAT1 could be a future direction to develop a novel therapeutic strategy to modulate and to enhance chemosensitivity to HDACi treatment.

## MATERIALS AND METHODS

### Cell lines and drug treatment

NPC cell lines, CNE1, CNE2 and C666–1 were obtained from the Cancer Institute of Southern Medical University, and evaluated whether cells were polluted with mycoplasma before the experimental process. Cells were cultivated in RMPI-1640 medium containing FBS (10%), penicillin (100 U/ml) and streptomycin (0.1 mg/ml) in a damp 5% CO_2_ atmosphere at 37 °C. The medium was renewed every other day, and cells at logarithmic growth stage were selected for subsequent experiments. Cells were seeded at a concentration of 1 × 10^5^ cells/well in 12-well plate 24 hours before treatment with HDACi (SAHA, Sigma-Aldrich, Shanghai, China). The selective HDAC inhibitors, MS-275 and PCI34051, and Bcl-2 inhibitors, ABT-737, ABT-263, and ABT-199, were purchased from Selleck Chemicals (Houston, TX). CAY10603 was purchased from Cayman Chemicals (Ann Arbor, MI).

### NPC specimens

NPC biopsies and nasopharyngectomized tissues used in this study were collected from 42 patients, who received radiation therapy in Affiliated Jinling Hospital, Medical School of Nanjing University. The Institutional Review Board of Affiliated Jinling Hospital, Medical School of Nanjing University has approved the collection and use of NPC samples in the study, and the patients provided written informed consent.

### MTS assay

Cells were inoculated in 96-well plates (1 × 10^4^ cells/well) were subjected to SAHA or other drugs for 24 hours. MTS assay was conducted with the MTS assay kit in accordance with the manufacturer’s prescription. Chemiluminescence was determined via Multi-label Counter. To ensure reproducibility, each assay was repeated for three times.

### Establishment of SAHA-tolerant NPC cell line

The SAHA concentration gradient progressive increase induction method was used to develop SAHA-resistant phenotype CNE1, CNE2, and C666-1 cell line. NPC cells (1 × 10^5^/ml) were seeded in non-SAHA medium for 1 day. Subsequently, the medium was renewed with the medium added SAHA (0.1 μmol/L) for 2 days, while removing the medium with the dead cells and drugs. Cells were then harvested and re-seeded in the medium without SAHA to recover before the beginning of another SAHA treatment. When cells adapted to the current SAHA treatment, we increased the SAHA concentration and repeated the treatment until cells exhibited tolerance to 2 μmmol/L SAHA. Both SAHA- sensitive and SAHA-resistant NPC cells were cultured under the same conditions and in drug-free medium for 2 days before commencing the experiments.

### Semi-quantitative RT-PCR

Total RNA was extracted with Purification Kit in accordance to the manufacturer’s specifications and the total RNA concentration and purity were determined with Nanodrop 2000 micro-volume spectrophotometer (Thermo Scientific, USA) by absorbance measurements. RT-PCR was performed on a PCR system with GAPDH as the internal reference. The primers used for gene amplification were: 5′-TTGTTCCAGAGCCCATGAT-3′ and 5′-TGAAAACCTTTACCCCAGGA-3′ for NEAT1; 5’-TTGGCCCCCGTTGCTT-3’ and 5’-CGGTTATCGTACCCCGTTCTC-3’ for Bcl-2; 5′-ACCACAGTCCATGCCATCAC-3′ and 5′-TCACCACCCTGTTGCTGTA-3′ for GAPDH. MiR-129 levels were determined by a stem-loop RT-qPCR, as previously described [36].

### Apoptosis analysis

Hoechst 33258 staining and subsequent microscopic visualization of concentrated chromatin and micro-nucleation were employed to investigate and evaluate the apoptosis of NPC cells. Furthermore, Annexin V/ PI staining was conducted via Annexin-Alexa Fluor 488 and PI [37]. The caspases 3/7 activity was analyzed using SensoLyte ® Homogeneous AFC Caspase - 3/7 Assay Kit as described by the manufacturer.

### Transfections of DNA plasmids

Transfection was conducted via Lipofectamine 2000 in accordance to the manufacturer’s specifications. A NEAT1 overexpression plasmid, pcDNA3.1-NEAT1, was commercially constructed by Genechem Co., Ltd. (Shanghai, China). The NEAT1 siRNA sequences used were 5′-GUGAGAAGUUGCUUAGAAACUUU-3′ and 5′-GGAAAGUUUCUAAGCAACUUCUCAC-3′. The siRNA for Bcl-2 was 5′-AACAUCGCCCUGUGGAUGACU-3′. The control counterparts were 5′-GUACCUGACUAGUCGCAGAAG-3′ and 5′-UCUGCGACUAGUCAGGUACGG-3′. All sequences were synthesized and bought from GenePharma. On the other hand, the miR-129-5p mimic, miR-129-5p antagonist, and controls were purchased from RiboBio [38].

### Pull-down assay

10^7^ C666-1 were collected, cleaved, and treated with ultrasound. Biotin labeled miR-129 and non-specific oligo probe were incubated with C-1 magnetic beads for 120 min at 25 °C to generate probe-covered beads. The resultant lysate with miR-129 or control probe was cultivated at 4 °C overnight. After washed with buffer, the RNA mix connected with the beads was eluted and isolated with RNeasy Mini Kit for PCR.

### Dual-luciferase reporter assay (DLR assay)

A wild-type and mutant NEAT1 fragment were established and introduced into downstream of the luciferase reporter gene of pMIR-REPORT plasmid as previously described [30]. The plasmid obtained was transfected to C666-4 cells via Lipofectamine 3000, followed by co-transfection with miR-129 mimic. Finally, DLR System Kit was applied to determine firefly and renilla luciferase activity. The Bcl-2 luciferase reporter assay was performed as previously described [19]. The sequences of Bcl-2 reporter construct oligonucleotides were: wild-type: 5′-CTAGTTCACTGTAGTTTGGTTTTATTTGAAAACCTGACAAAAAAAAAGTTCCAGGT-3′ and 5′-AAACACCTGGAACTTTTTTTTTGTCAGGTTTTCAAATAAAACCAAACTACAGTGA-3′; mutant: 5′-CTAGTTCACTGTAGTTTGGTTTTATTTGAAAACCTGA*TAGACA*AAAAGTTCCAGGT-3′ and 5′-AAACACCTGGAACTTTTTGTCTATCAGGTTTTCAAATAAAACCAAACTACAGTGA-3′.

### Western blotting

Western Blotting (WB) was conducted according to previous protocols. Antibodies intended for cleaved caspases 3, Bcl-2, Bcl-XL, Mcl-1, and Actin, were bought from Cell Signaling (Danver, MA, USA).

### Xenografts

All animal experiments have been approved by the Animal Care and Use Committee of Affiliated Jinling Hospital, Medical School of Nanjing University. 35- to 42-day-old Nu/Nu female mice were confined in micro isolator cages in aseptic conditions and provided with sufficient water and food. Xenograft tumors were established by subcutaneously injecting mice with 4 × 10^6^ of C666-1R cells. Seven days after tumor inoculation, mice were subjected to either SAHA (50 mg/kg/day) daily for 14 consecutive days or the control vehicle, or intratumor injection of control or miR-129 (100 μl, 2 mg/ml) as described previously [38]. For the ABT-199 and SAHA combination treatment, the mice were subjected to either SAHA (50 mg/kg/day) or the control vehicle by intraperitoneal (I.P.) injection, or control or ABT-199 (50 mg/kg/day) by oral gavage daily for 14 consecutive days. Calipers were used to quantify tumor volumes, which were then calculated by the formula 1/2 × length × width^2^. When tumor size reached 1.0 cm^3^, mice were euthanized, and tumors were dissected, fixed with 10% formalin and embedded in paraffin. TUNEL immunostaining was conducted on 5-mm tumor slices. Signals were detected through Alexa Fluor 488–conjugated secondary antibody with nuclear counterstaining by DAPI.

### Statistical analysis

Statistical analysis was performed with GraphPad Prism 5.0. Kaplan–Meier method and the T-test analysis. A value of P < 0.05 was considered as statistically significant.

## Supplementary Material

Supplementary Figures
