# Supplementary material for: LncRNA NEAT1/miR-129/Bcl-2 signaling axis contributes to HDAC inhibitor tolerance in nasopharyngeal cancer
Source: Aging (Albany NY). 2020 Jul 21;12(14):14174–88. doi: 10.18632/aging.103427 (PMC7425502; doi:10.18632/aging.103427)
Supplement: Supplementary Figures [file aging-12-103427-s001..pdf]

SUPPLEMENTARY FIGURES

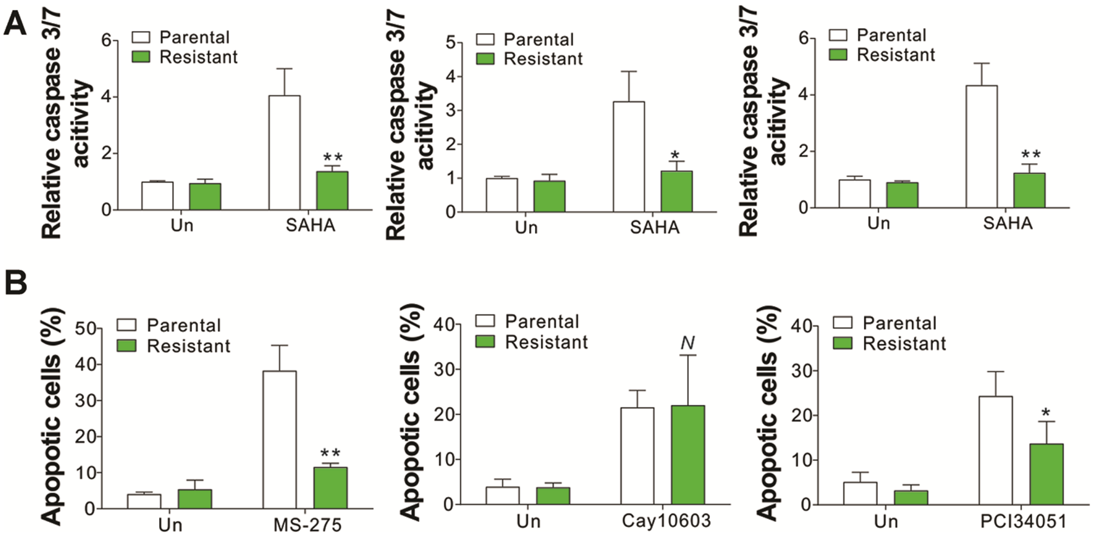

Supplementary Figure 1. Less apoptosis was induced the SAHA resistant NPC cells. (**A**) The caspase-3/7 activity in parental and SAHA-tolerant cells subjected to 4 μmol/L of SAHA for 24 h. (**B**) The apoptosis of parental and SAHA-tolerant C666-1 cells subjected to 2.5 μmol/L of MS-275, 4 nmol/L of Cay10603, or 10 μmol/L of PCI34051 for 24 h. Each experiment was performed for 3 times. N, p>0.05; *, p<0.05; **, p<0.01.

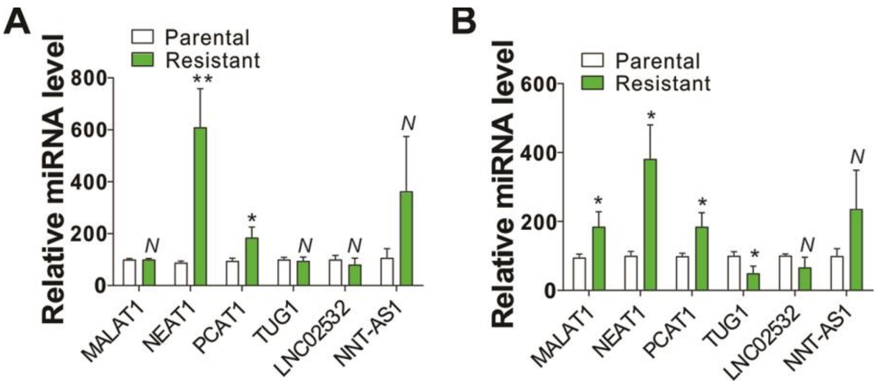

Supplementary Figure 2. NEAT1 expression was induced in SAHA resistant NPC cells. (**A**) The expression of indicated LncRNA in CNE-1 parental and resistant cells. (**B**) The expression of indicated LncRNA in CNE-2 parental and resistant cells. Each experiment was performed for 3 times. N, p>0.05; *, p<0.05; **, p<0.01.

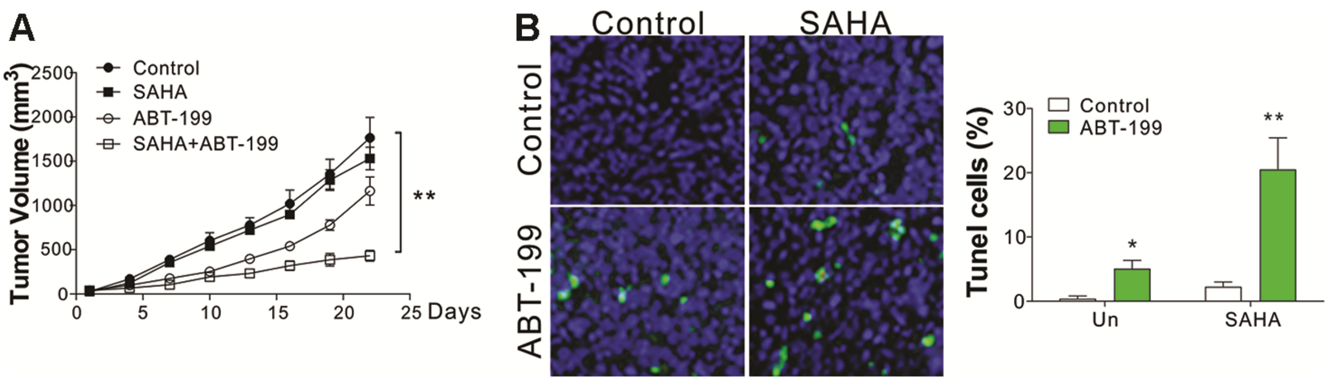

Supplementary Figure 3. ABT-199 enhanced the killing effect of SAHA *in vivo*. (**A**) The tumor growth curves of nude mice xenografted with C666-1R cells and treated with SAHA and/or ABT-199. (**B**) The TUNEL staining of tumors in each group. *, $p<0.05$; **, $p<0.01$.
